# QTc prolongation and torsades de pointes (TdP) in individuals undergoing methadone maintenance treatment (MMT): A systematic review and meta-analysis

**DOI:** 10.1097/MD.0000000000045304

**Published:** 2025-10-24

**Authors:** Mohammad Hossein Paknahad, Azam Soleimani, Fatereh Baharlouei Yancheshmeh, Parisa Adib-Hajbagheri, Atefeh Amerizadeh, Raheleh Karimi

**Affiliations:** a Cardiology Department, Abadan University of Medical Science, Abadan, Iran; b Cardiac Rehabilitation Research Center, Isfahan Cardiovascular Research Institute, Isfahan University of Medical Sciences, Isfahan, Iran; c Echocardiography Department, Chamran Cardiovascular Medical and Research Hospital, Isfahan University of Medical Sciences, Isfahan, Iran; d Department of Biostatistics and Epidemiology, School of Health, Isfahan University of Medical Sciences, Isfahan, Iran.

**Keywords:** arrhythmia, cardiovascular, methadone, QTc prolongation, torsades de pointes

## Abstract

**Background:**

The incidence of corrected QT interval (QTc) prolongation and torsades de pointes (TdP) in individuals undergoing methadone maintenance treatment (MMT) is a significant concern, as studies indicate that methadone can lead to these cardiac complications.

**Methods:**

A systematic search was performed across various databases including PubMed, Scopus, and Embase from January 2000 to July 2025. Risk of bias was assessed using the Risk Of Bias In Non-randomized Studies of Interventions (ROBINS-I) for observational studies.

**Results:**

Twenty-two observational studies were included. The pooled mean age of patients undergoing MMT across the included studies was 40.8 years (95% confidence interval [CI]: 37.9–43.8). The overall pooled proportion of male participants across the included studies was 73% (95% CI: 66–81%). Incidence of QTc prolongation among patients on MMT showed a prevalence of 34% (95% CI: 24–43%). The pooled incidence of TdP was 2% (95% CI: 0–5%) after removing outlier study. The majority of evidence supports a dose-dependent relationship between methadone and QTc prolongation, though TdP remains rare and inconsistently linked to dose. This underscores the need for individualized dose titration and electrocardiogram monitoring, especially at higher daily doses (>100 mg).

**Conclusion:**

MMT is associated with a substantial risk of QTc prolongation and a low but clinically relevant risk of TdP. Careful dose adjustment and regular electrocardiogram monitoring, particularly at higher doses, are essential to minimize cardiac complications.

## 1. Introduction

Methadone is a long-acting opioid agonist with an elimination half-life of 24 to 36 hours and is widely used to treat opiate and heroin addiction via methadone maintenance therapy (MMT), which is the most common treatment modality for treatment of opioid dependence.^[1–3]^ The effectiveness of MMT in reducing opioid use and maintaining treatment is well established.^[4–7]^ However, MMT has a critical safety concern in that the therapeutic dose is very close to its toxic dose.^[8]^ The ideal daily oral maintenance dose is usually between 60 and 100 mg.^[9,10]^ Methadone dose appears to be a critical component in corrected QT interval (QTc) prolongation.^[11–13]^

QTc prolongation and torsades de pointes (TdP) are known cardiac side effects seen more in MMT subjects.^[10,12,14,15]^ Methadone, unlike other opioids, acts primarily through blockade of the cardiac human Ether-à-go-go related gene potassium channel which is responsible for the rapid component of the delayed rectifier potassium current in the cardiomyocyte, which causes a delay in cardiac repolarization.^[12,16–18]^ Drug-induced severe QTc prolongation (>500 ms) is a definitive risk factor of TdP ventricular tachycardia and sudden death.^[19,20]^ The risk of sudden cardiac death increases 4-fold when QTc is ≥500 ms (milliseconds).^[21,22]^

Previous reviews,^[11,12]^ have studied the interaction of methadone, morphine, oxycodone, fentanyl, and buprenorphine use with ventricular arrhythmias. These studies have generally considered a broad range of arrhythmias, such as ventricular premature beats (PVCs), ventricular tachycardia, ventricular fibrillation, and TdP but without reporting pooled incidence estimates or specifically isolating the effects of methadone. In contrast, our systematic review and meta-analysis focused exclusively on methadone, providing pooled estimates of QTc prolongation and TdP in patients undergoing MMT.

## 2. Materials and methods

The preferred reporting items for systematic reviews and meta-analyses statement, published in 2021 was used as guideline for this study. The protocol for this review has not been registered with any organization. All the steps including searching, selection of included papers, and quality assessment of articles were performed by 2 authors independently, and any discrepancies were resolved through discussion or consultation with a third reviewer.

### 2.1. Search strategy

A comprehensive literature search was performed across PubMed, Embase, Scopus, Web of Science, and Cochrane CENTRAL from January 2000 to July 2025. The search was limited to English-language studies involving human subjects. The strategy combined controlled vocabulary (e.g., MeSH/Emtree) and free-text terms, covering three main domains: methadone treatment, QTc prolongation, TdP, cardiac arrhythmia incidence. Searches were adapted to fit the syntax and indexing of each database. Boolean operators (AND, OR) were used to combine concepts. Truncation (e.g., “prolong*”) was used when applicable to capture word variants. The detailed search strategy is provided in Appendix 1, Supplemental Digital Content, https://links.lww.com/MD/Q391.

### 2.2. Inclusion and exclusion criteria

Studies reporting the incidence or prevalence of QTc prolongation or TdP in individuals on MMT were included. Eligible studies encompassed observational studies (cohort and cross-sectional). Case reports were excluded from this analysis due to their small sample sizes. Studies reported QTc prolongation or TdP due to methadone overdose were excluded.

Randomized clinical trials on MMT-induced QTc prolongation and TdP are lacking due to ethical, methodological, and practical challenges. Exposing participants to a known risk of serious arrhythmia would be unethical, particularly when safer alternatives exist. Additionally, TdP is rare, making randomized clinical trials statistically inefficient for assessing its incidence.

### 2.3. PICO

Population: individuals/patients on methadone maintenance treatment (MMT).

Intervention: MMT.

Outcomes: QTc prolongation; long QT; TdP.

Context/measurement: electrocardiogram (ECG/EKG); QT interval.

### 2.4. Data extraction

By carefully reading the full text of the included studies data were extracted including first author’s name, year, study design, sample size, percentage of the male participants, and MMT duration. Outcomes that extracted were mean of QTc, incidence of QTc prolongation, and incidence of TdP after MMT.

### 2.5. Ethical considerations

All results and analyses were from previous published studies; thus, no ethical approval and patient consent are required. All the included studies had had ethical approval statements.

### 2.6. Statistical method

We conducted data extraction from each article to obtain the number of cases in QTc longer than 500 ms, QTc lower than 500 ms, and TdP. Also, mean and standard deviation of age and QTc were extracted. The overall effect size was reported as the proportion or mean along with a 95% confidence interval (CI).

Heterogeneity among studies was assessed using both the *I*^2^ statistic and the *Q* statistic. In cases where heterogeneity was present (*I*^2^ > 50 and *P* < .05) we utilized a random-effects model to combine the study results. The Supplementations File, Supplemental Digital Content, https://links.lww.com/MD/Q390 provide funnel plots for heterogeneity. Conversely, in the absence of significant heterogeneity, a fixed-effects model was employed. To evaluate publication bias, we utilized the Egger’s regression test and applied the “trim and fill” method if any significant publication bias was detected.^[23]^ The sensitivity analysis was measured for assessment of the robustness of the combined risk estimates to evaluate whether the low-quality studies would influence the overall result. All statistical analyses were conducted using Stata version 14.

## 3. Results

### 3.1. Search

**Figure 1.**
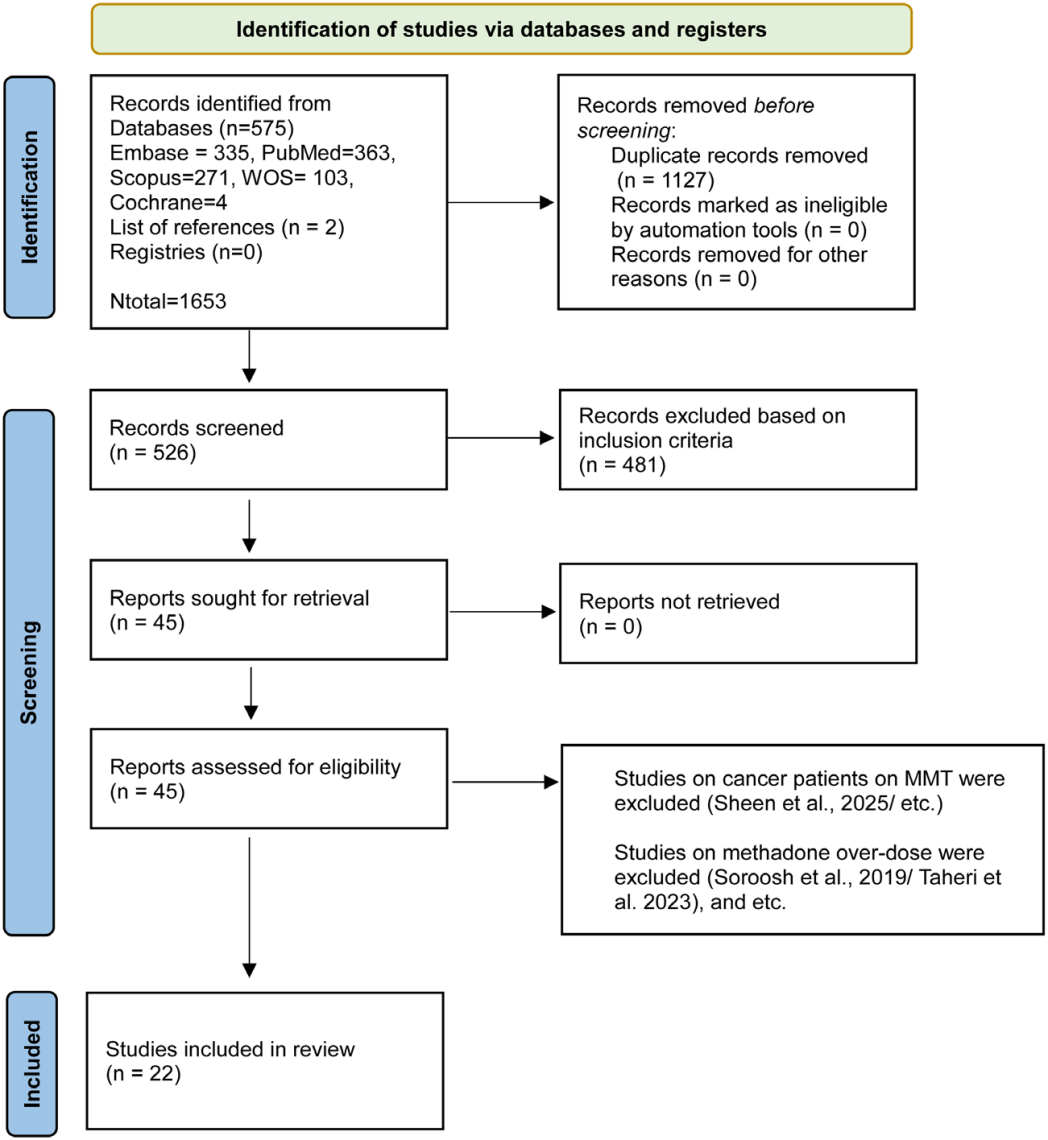
PRISMA flow diagram for the present systematic review. PRISMA = preferred reporting items for systematic reviews and meta-analyses.

The flowchart of study selection process was depicted in Figure 1. Our primary search resulted in 645 articles. After removing duplications 308 articles remained. Next the articles were screened based on their titles, abstracts, study design, presence of enough data, and other criteria and finally 22 studies were included.^[14,24–41]^ Except 2 studies that their population were war causalities,^[29]^ and veterans,^[33]^ others were previously addicted subjects. Table 1 showed the summary and extracted data of the included studies.

**Table 1:** Summary and extracted data of the included studies.

First authors/year	Mean age (yr)	Patients on MMT	Male no & percentage	MMT duration	Median daily dosage mg/d	QTc > 450 ms	(TdP)	Dose association with QTc prolongation
Mayet 2011^[37]^	39.9 ± 8.25	155	109 (70.8% M)	25 mo	84.9	18.1% (n = 15) 0–2% > 500	0%	Linear regression found total daily methadone dose (β = 0.318, *P* = .003) and stimulant use (β = −0.213, *P* = .043) predictive of QTc length
Chang 2012^[25]^	37.8 ± 7.5	283	229 (84% M)	6 mo	40	60.70%	0%	(*R* = 0.201, *P* = .0007) Significant in males (*R* = 0.210, *P* = .0014) but not in females (*R* = 0.164, *P* = .2363)
Cruciani 2005^[26]^	45.3 ± 9.4	104	63 (61% M)	12.5 mo	110	32% (n = 33) 0 > 500 ms	0%	Receiving methadone for <12 mo had a strongly positive relationship between dose and QTc (Spearman ρ = 0.60, *P* =.01, d = 1.5)
Demarie 2011^[27]^	38.2 ± 0.0	190	153 (80% M)	2 wk	60	13.5% (n = 25) 10 > 480 ms	0%	In 37% of cases, QTc normalized after reducing methadone to 50 mg; 10 patients with QTc > 480 ms received treatment, and 1 case was linked to ischemia
Krantz 2008^[35]^	–	151	–	6 mo	10–30	76%	0%	–
Esfahani 2012^[29]^	45.6 ± 6.1	100	100 (100% M)	1–108 mo	20–140	25%	0%	No significant relationship was observed between QTC interval and methadone dose (*R* = 0.025, *P* = .8), duration of treatment (*R* = −0.048, *P* = .68)
Fanoe 2007^[30]^	41.3 ± 8.3	407	300 (64% M)	7 yr (0.5;20)	100 (50;235)	28%	0%	Methadone dose was associated with longer QT interval of 0.140 ms/mg (*P* = .002). A 50 mg higher methadone dose was associated with a 1.2 (95% CI 1.1–1.4) times higher odds for syncope
Anchersen 2009^[24]^	42 ± 7	173	120 (69.36% M)	47 mo	111	15% (n = 26) 4.6% (n* *= 8) > 500	0%	Positive correlation both in Pearson’s correlation (*R* = 0.37, *P* < .01) and in the multiple linear regression analysis (*B* = 0.37, *P* < .01).
Katz 2013^[34]^	41.2 ± 13.1	568	322 (57.44% M)	43 d	100–120	195 (43.6 %) > 450 < 500 21 (47.6%) > 500	0%	In 9 patients with marked QTc prolongation, dose reduction (mean = 22 mg/d) correlated with QTc change (*R*^2^ = 0.20, *P* = .057), but not significantly
Hassamal 2015^[33]^	56.96 ± 6.48	49	47 (96% M)	8.72 ± 4.50 yr	78.20 ± 25.30	9 (18%)>450 3 > 475 < 500 2 > 500	0%	Methadone dose was significantly higher in those with QTc change ≥ 24 ms (88.48 ± 27.20 mg vs 68.96 ± 19.84 mg)
Maremmani 2005^[36]^	34 ± 6	83	(75.9% M)	6 mo	10–600	83% 2 > 500	0%	No correlation emerged between QTc values and methadone dosages
Fareed 2013^[31]^	59 ± 9	55	51 (94% M)	5 yr	98 ± 55	41–56% > 450 4–10% > 500	0%	–
Peles 2007^[38]^	37 ± 9	138	98 (71% M)	2 ± 0.4 yr	170.9	3 (2.7%)	0%	–
Pearson 2005^[14]^	46 ± 11	5503	59 (39% M)			27%	73.0%	–
Perrin-Terrin 2011^[39]^	34 ± 8	42	37 (88% M)	3 yr	45	5 (11.9%) 0 > 500	3 9.52%	Methadone can prolong the QT interval at dose usually used for MMT in France
Chang 2022^[42]^	40.9 ± 0.7	154	118 (76.6% M)	3 mo	63.8 ± 3.3	24.70%	-	Methadone dose interacted significantly with rs11911509 genotype (*P* = .01), with QTc prolongation seen only in AA carriers
Deuss 2024^[43]^	36.2 ± 7.4	45	32 (71.11% M)	1 mo, 6 mo	50–150 (1 mo) 60–120 6 mo	0%	0%	QTc was not associated with the serum concentrations of methadone
Ehret 2006^[28]^	37	167	110 (66% M)	5 yr	100	16.2% (n = 27)	3.6% (n = 6)	Higher daily methadone dose showed a weak but significant association with QTc prolongation (rs = 0.20; *P* < .01)
Santin 2023^[40]^	42 ± 11.1	165	134 (81.2% M)	10 yr	80	14% (n = 23)	0%	The dose of methadone was the only independent risk factor for QTc prolongation in the multivariate analyses: the QTc increased by 28 ms when the dose of MTD increased from 50 to ≥150 mg/d
Titus-Lay 2021^[41]^	36 ± 12	93	25 27% M	≥28 d–5 yr	98 ± 55	15.1% (n = 14)	0%	No correlation emerged between QTc values and methadone dosages
Gheshlaghi 2013^[44]^	37.7 ± 8.1	122	103 84% M	4 wk	42.5 ± 22.2	-	0%	A significant positive correlation between methadone dose and the age-adjusted QTc interval in total (*R* = 0.4558, *P* < .001) and in males (*R* = 0.6320, *P* < .001), but a negative correlation in females (*R* = 0.5348, *P* = .018)
Stallvik 2013^[45]^	32.4 ± 8.5	90	90 (100%)	2 mo	0–59 60–109 110–150	–	0%	Patients receiving methadone over 60 mg/d are at a risk of greater QTc prolongation
Zerdazi 2019^[46]^	47.7 ± 9.3	302	248 82.1%	4–7 yr		14 (12.5%) 2 (1.8%) > 500		There was also an influence of methadone dosage (*P *< .001). multivariate model, also shows the effect of methadone dosage (*P *< .001)

CI = confidence interval, ms = milliseconds, MMT = methadone maintenance treatment, QTc = corrected QT interval, TdP = torsades de pointes.

### 3.2. Age

**Figure 2.**
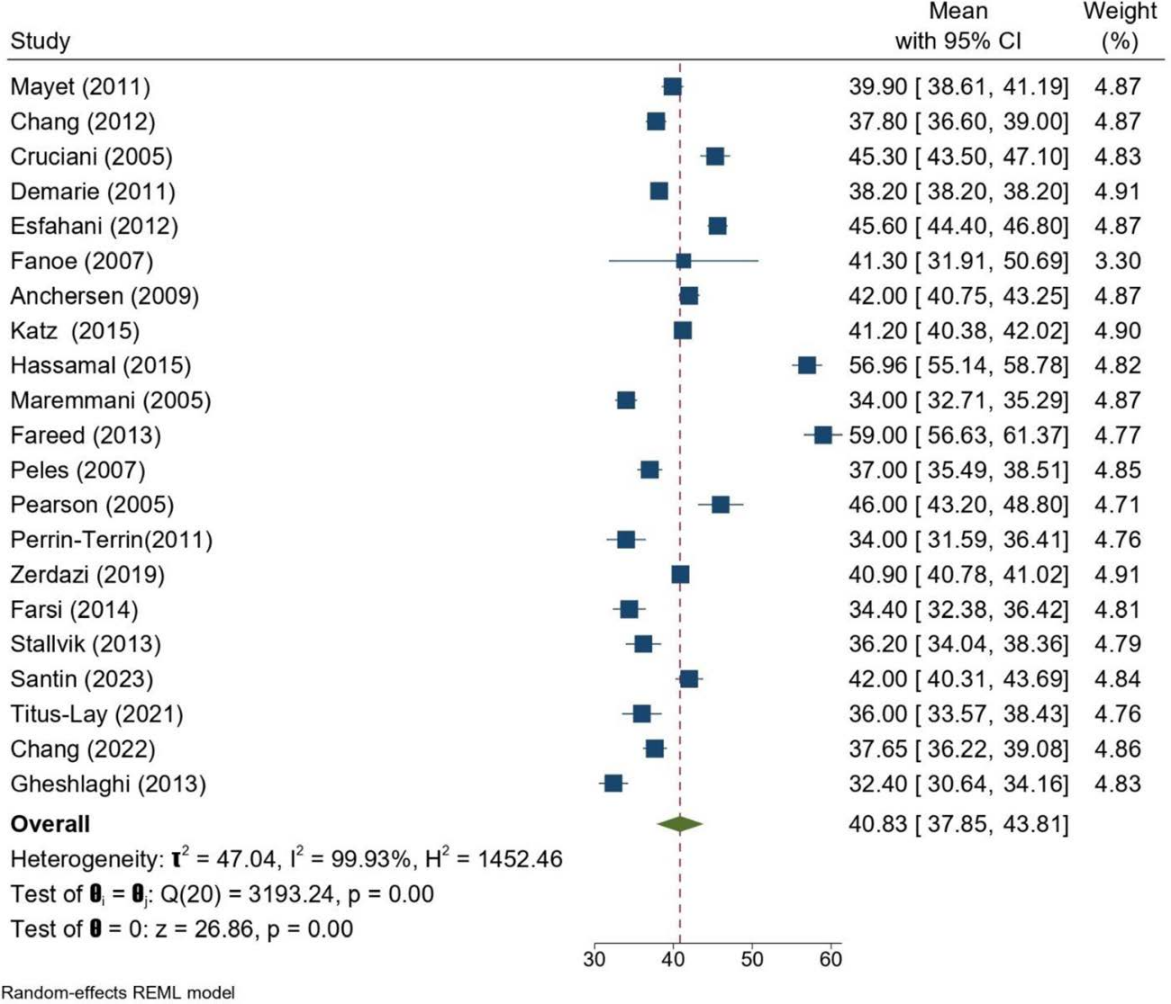
Forrest plot of mean age of patients undergoing MMT across the included studies. CI = confidence interval, MMT = methadone maintenance treatment, REML = restricted maximum likelihood.

The pooled mean age of patients undergoing MMT across the included studies was 40.8 years (95% CI: 37.9–43.8). Considerable heterogeneity was observed among studies (*I*^2^ = 99.9%, *P* < .001), reflecting differences in study populations and treatment settings. Despite this variation, most studies reported mean ages in the late 30s to mid-40s, consistent with the typical demographic profile of patients in MMT program (Fig. 2).

### 3.3. Sex

**Figure 3.**
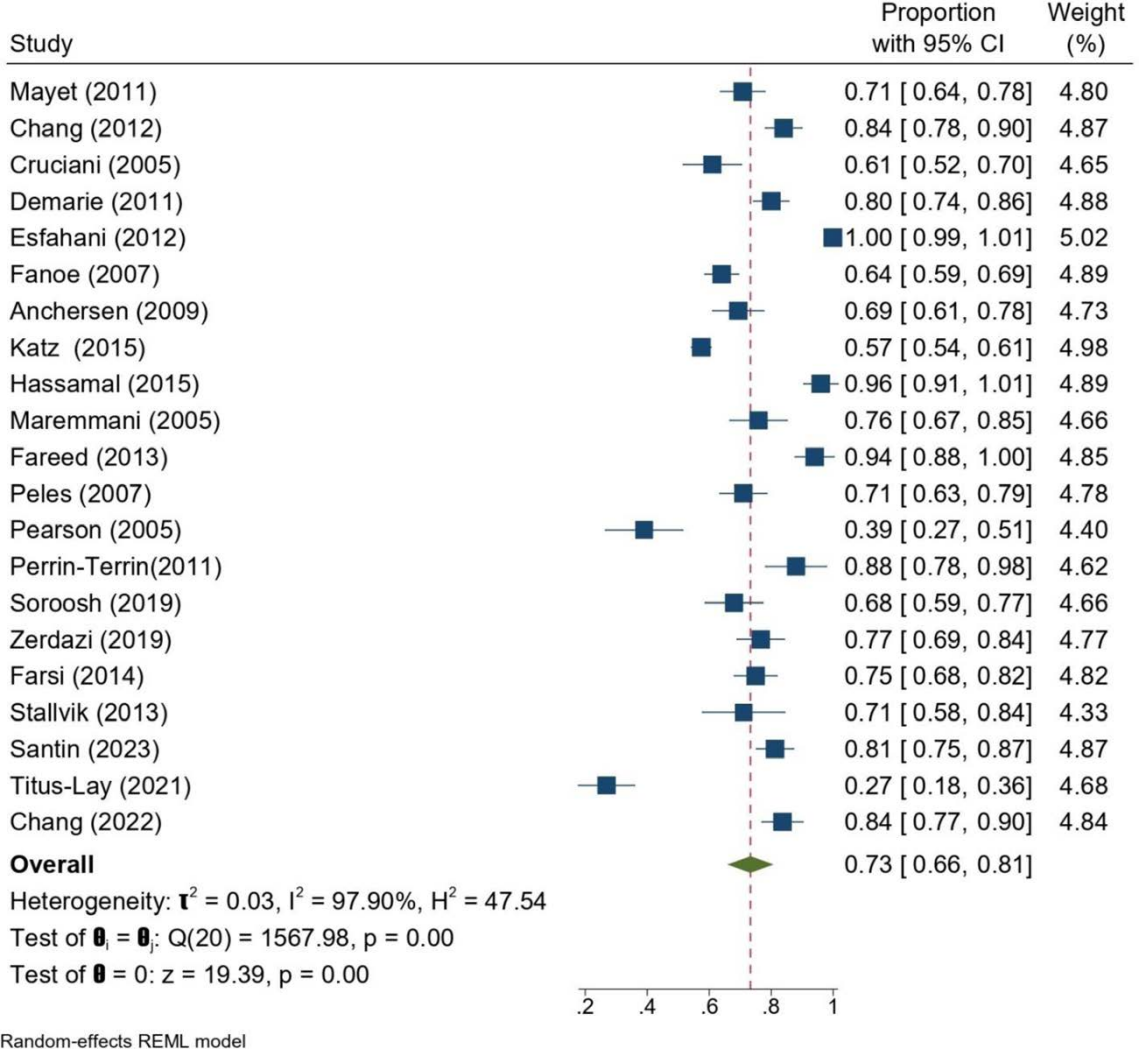
Forrest plot of sex (male %) of patients undergoing MMT across the included studies. CI = confidence interval, MMT = methadone maintenance treatment.

The overall pooled proportion of male participants across the included studies was 73% (95% CI: 66–81%). Substantial heterogeneity was observed (*I*^2^ = 97.9%, *P* < .001), likely due to differences in sample sizes, geographic regions, and recruitment strategies. Nonetheless, the majority of cohorts demonstrated a clear predominance of men, reflecting the higher prevalence of opioid dependence and enrollment in MMT programs among males (Fig. 3).

### 3.4. Meta-analysis

#### 3.4.1. QTc prolongation

**Figure 4.**
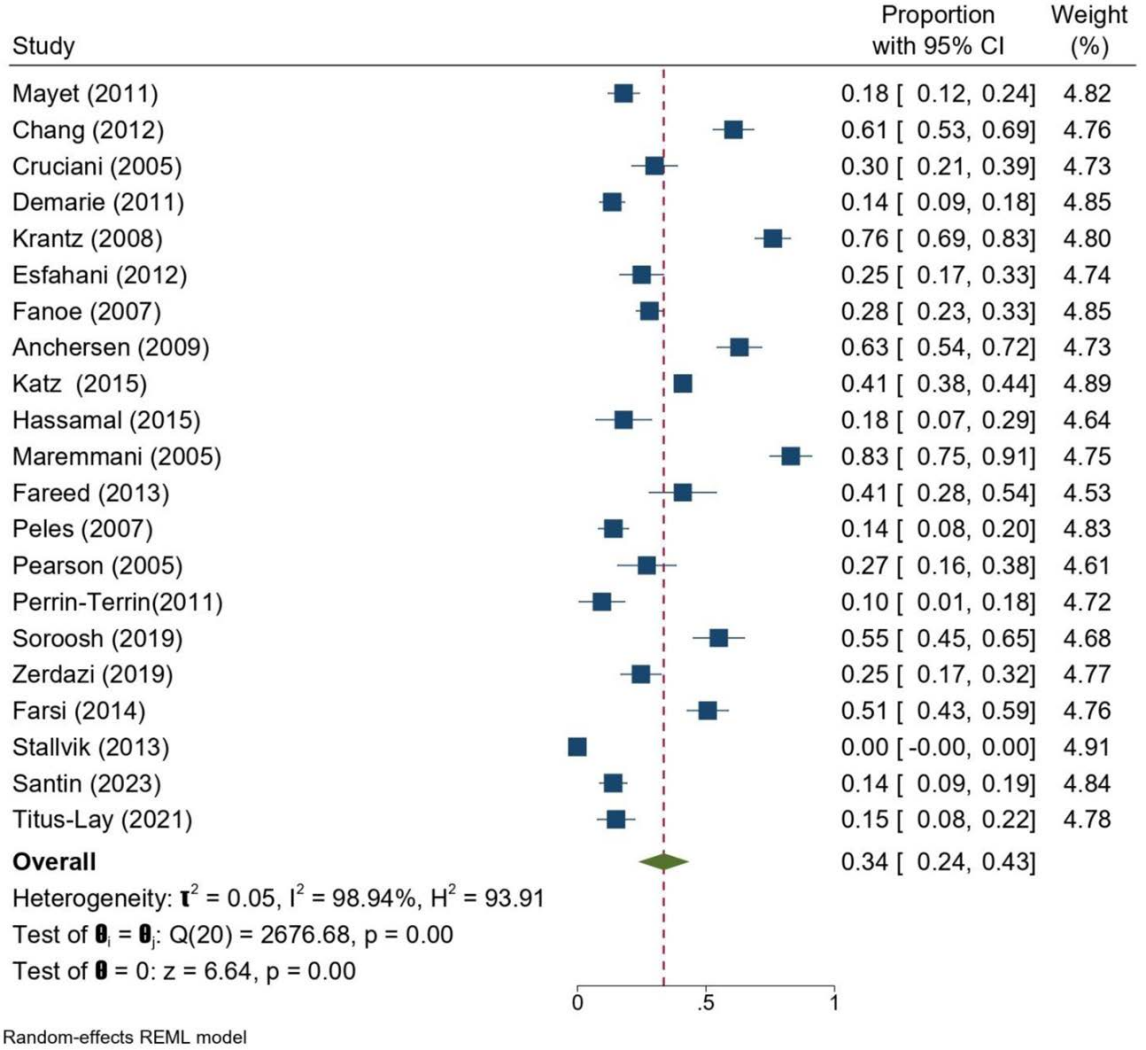
Forrest plot of QTc prolongation of patients undergoing MMT across the included studies. CI = confidence interval, MMT = methadone maintenance treatment, QTc = corrected QT interval.

The pooled analysis of studies reporting QTc prolongation among patients on MMT showed a prevalence of 34% (95% CI: 24–43%). Heterogeneity across studies was high (*I*^2^ = 98.9%, *P* < .001), reflecting differences in population characteristics, methadone dosages, and follow-up durations. Despite this variability, a significant proportion of patients developed mild-to-moderate QTc prolongation, underscoring the importance of continuous cardiac monitoring even in the absence of severe prolongation (Fig. 4).

#### 3.4.2. TdP

**Figure 5.**
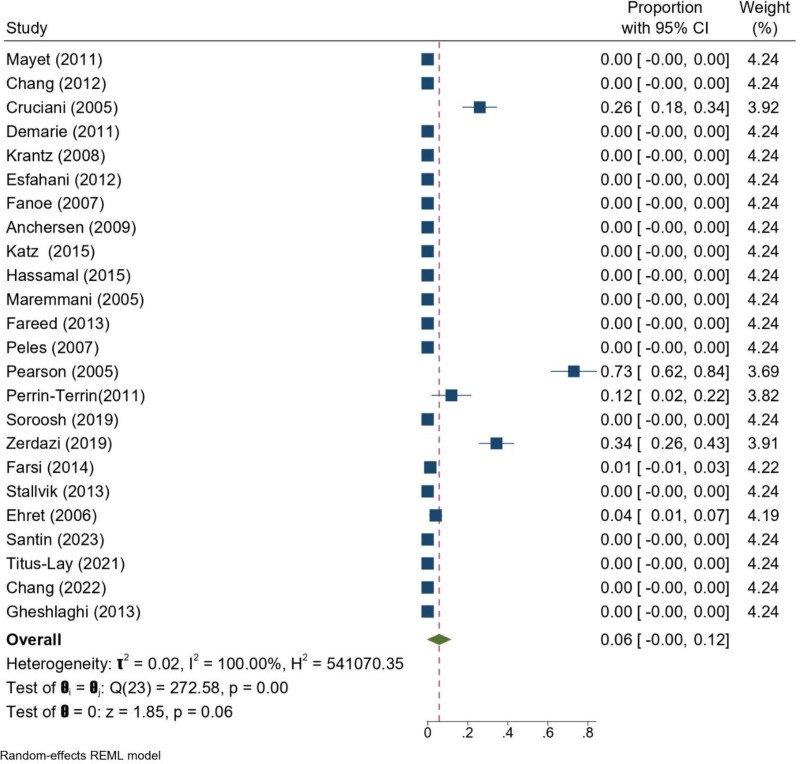
Forrest plot of TdP of patients undergoing MMT across the included studies. CI = confidence interval, MMT = methadone maintenance treatment, TdP = torsades de pointes.

**Figure 6.**
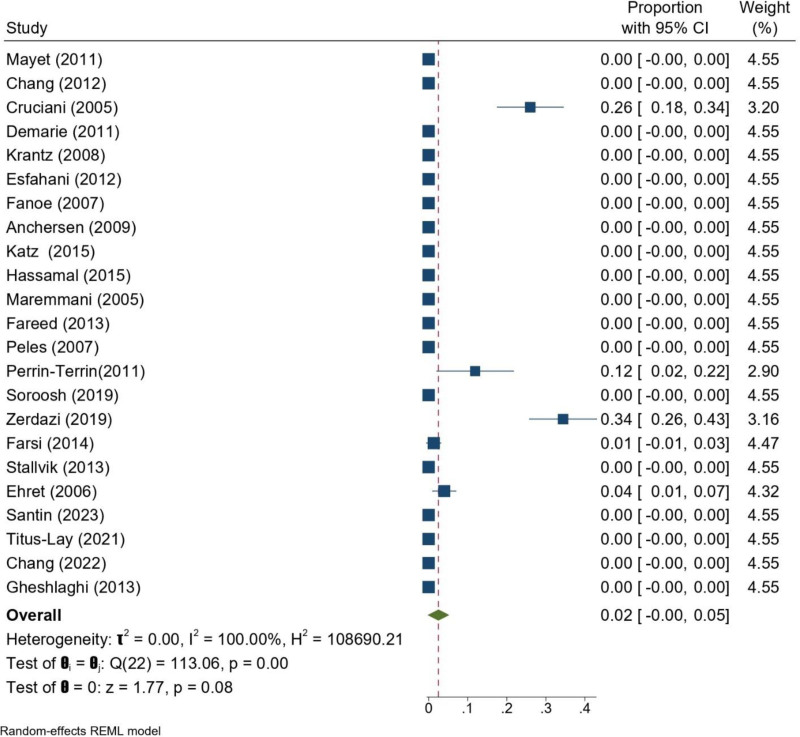
Forrest plot of TdP of patients undergoing MMT across the included studies excluding. Pearson et al.^[14]^ CI = confidence interval, MMT = methadone maintenance treatment, TdP = torsades de pointes.

The pooled analysis of studies, including Pearson et al,^[14]^ estimated a TdP prevalence of 6% (95% CI: 0–12%; Fig. 5). When the Pearson study was excluded, the pooled incidence decreased to 2% (95% CI: 0–5%), although this result did not reach statistical significance (*P* = .08). The latter estimate was substantially lower, highlighting the influence of Pearson et al’s pharmacovigilance-based data on the overall outcome. In both analyses, heterogeneity remained high (*I*^2^ = 100%), largely driven by a small number of studies that reported TdP events, while most reported none. Taken together, these findings indicate that TdP is an uncommon but clinically important complication of methadone treatment (Fig. 6).

### 3.5. Risk of bias assessment

**Figure 7.**
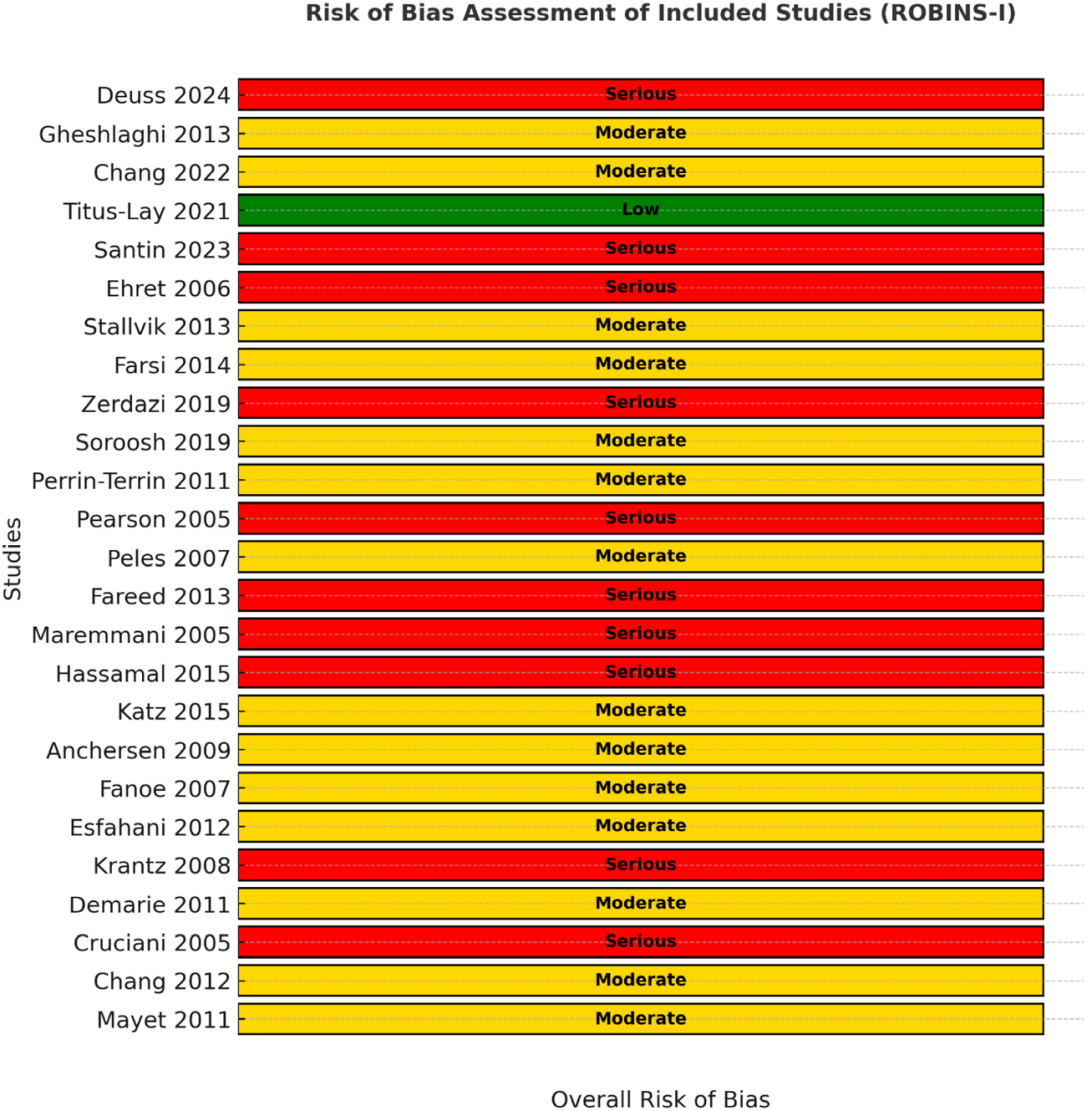
Risk of bias summary. ROBINS-I = Risk Of Bias In Non-randomized Studies of Interventions.

Risk of bias assessment using the Risk Of Bias In Non-randomized Studies of Interventions (ROBINS-I) for observational studies showed that cross-sectional studies were generally rated as having a *moderate* risk of bias, primarily due to confounding risk of bias and the inability to establish temporality. Retrospective cohort studies were more vulnerable to confounding, selection bias, and missing data, and therefore were often rated as *serious* risk. In contrast, the prospective study by Titus-Lay et al^[41]^ demonstrated the lowest risk of bias and was rated as *low*. The study by Pearson et al,^[14]^ based on Food and Drug Administration (FDA) pharmacovigilance reports, was considered to have a *serious* risk of bias due to inherent selection bias and limitations in outcome measurement. Table 2 and Figure 7 showed the summary of risk of bias assessment.

**Table 2:** Risk of bias assessment.

First author/year	Study design	Bias due to confounding	Bias in selection of participants	Bias in classification of interventions	Bias due to deviations from intended interventions	Bias due to missing data	Bias in measurement of outcomes	Bias in selection of reported results	Overall risk of bias
Mayet 2011	Cross-sectional	Moderate	Low	Low	Low	Low	Moderate	Low	Moderate
Chang 2012	Cross-sectional	Moderate	Low	Low	Low	Low	Moderate	Low	Moderate
Cruciani 2005	Cross-sectional	Serious (confounding, small n)	Low	Low	Low	Low	Moderate	Low	Serious
Demarie 2011	Cross-sectional	Moderate	Low	Low	Low	Low	Moderate	Low	Moderate
Krantz 2008	Retrospective cohort	Serious	Low	Low	Low	Moderate	Moderate	Low	Serious
Esfahani 2012	Retrospective cohort	Moderate	Low	Low	Low	Moderate	Moderate	Low	Moderate
Fanoe 2007	Cross-sectional	Moderate	Low	Low	Low	Low	Moderate	Low	Moderate
Anchersen 2009	Cross-sectional	Moderate	Low	Low	Low	Low	Moderate	Low	Moderate
Katz 2015	Cross-sectional	Moderate	Low	Low	Low	Low	Low	Low	Moderate
Hassamal 2015	Retrospective cohort	Serious (veterans only)	Moderate	Low	Low	Low	Moderate	Low	Serious
Maremmani 2005	Retrospective cohort	Serious (heterogeneous doses)	Low	Low	Low	Low	Moderate	Low	Serious
Fareed 2013	Retrospective cohort	Serious (long-term follow-up)	Low	Low	Low	Moderate	Moderate	Low	Serious
Peles 2007	Cross-sectional	Moderate	Low	Low	Low	Low	Low	Low	Moderate
Pearson 2005	Retrospective cohort	Serious (FDA reports)	High (selected cases)	Low	Low	Low	Serious	Low	Serious
Perrin-Terrin 2011	Cross-sectional	Moderate	Low	Low	Low	Low	Moderate	Low	Moderate
Soroosh 2019	Cross-sectional	Moderate	Low	Low	Low	Low	Moderate	Low	Moderate
Zerdazi 2019	Retrospective cohort	Serious (gene-dose interaction, limited generalizability)	Low	Low	Low	Low	Moderate	Low	Serious
Farsi 2014	Cross-sectional	Moderate	Low	Low	Low	Low	Moderate	Low	Moderate
Stallvik 2013	Cross-sectional	Moderate	Low	Low	Low	Low	Moderate	Low	Moderate
Ehret 2006	Retrospective cohort	Serious	Low	Low	Low	Low	Moderate	Low	Serious
Santin 2023	Retrospective cohort	Serious	Low	Low	Low	Low	Moderate	Low	Serious
Titus-Lay 2021	Prospective	Low	Low	Low	Low	Low	Low	Low	Low
Chang 2022	Retrospective cohort	Moderate	Low	Low	Low	Low	Moderate	Low	Moderate
Gheshlaghi 2013	Comparative observational	Moderate	Low	Low	Low	Low	Moderate	Low	Moderate
Deuss 2024	Retrospective cohort	Serious (real-world, EHR-based)	Low	Low	Low	Low	Moderate	Low	Serious

EHR = electronic health record, FDA = Food and Drug Administration.

## 4. Discussion

To the best of our knowledge, this is the first systematic review and meta-analysis to evaluate the incidence of QTc prolongation and TdP in patients undergoing MMT. Across 22 observational studies, our pooled analysis showed that 34% of patients experienced QTc prolongation, whereas only 2% to 6% developed TdP.

Of the 22 observational studies included, 18 specifically addressed the association between methadone dose and QTc prolongation or change in TdP, but meta-analysis was precluded because variable reporting formats were used, with some reporting odds ratios/hazard ratios and others providing only descriptive outcomes. Of these 18 studies, the majority, approximately two-thirds, identified a clear dose–response relationship, showing that higher methadone doses were highly correlated with increased QTc. For example, Fano et al^[30]^ reported an increase of 0.14 ms/mg of methadone (*P* = .002), Anchersen et al^[24]^ found a positive correlation (*R* = 0.37, *P* < .01), and Santin et al^[40]^ found methadone dose to be the only independent factor for QTc prolongation, with QTc increasing by 28 ms as the daily dose increased from 50 to ≥150 mg. The same correlation was found by Chang et al ^[42]^ in men (*R* = 0.6320, *P* < .001), while Deuss et al demonstrated an independent effect of methadone dose in multivariate models (*P* < .001).^[43]^ According to Gheshlaghi et al methadone causes dose-dependent QTc prolongation, with doses under 60 mg/d posing lower cardiac risk. Since such low doses may not be suitable for all patients, regular ECG monitoring is recommended, particularly for those on higher methadone doses.^[44]^ On the other hand, smaller studies^[29,36,41,45]^ failed to show a significant correlation, possibly due to differences in methods, small sample sizes, inconsistent dosing schedules, or short follow-up. Katz et al^[34]^reported a trend toward dose-proportional improvement in QTc after dose reduction, but this was not statistically significant. For TdP, all but a handful of studies reported no events, and even those that did^[26,39]^ found no reliable dose–response relationship. Pearson et al^[14]^ had an unusually high rate of TdP, but as this was an FDA drug surveillance report and not a well-defined clinical cohort, it is considered an outlier due to severe reporting bias. Overall, the findings suggest that although TdP is rare and heterogeneously related to methadone dose, QTc prolongation is often dose-dependent and therefore dose titration and cautious ECG monitoring, particularly at doses >100 mg/d, should be recommended.

In the broader pooled analysis, the estimated prevalence of TdP among MMT patients was 6% (95% CI: 0–12%). This higher estimate was largely influenced by the study of Pearson et al,^[14]^ which reported an unusually high TdP prevalence based on FDA spontaneous reporting data. Because this source reflects pharmacovigilance reports rather than a defined patient cohort, it may substantially overestimate the true incidence due to reporting bias. In contrast, when Pearson et al’s study was omitted, the pooled TdP incidence dropped to 2% (95% CI: 0–5%), with most studies reporting no TdP events at all. This suggests that while TdP is a rare but serious complication of MMT, its true prevalence is likely closer to the conservative estimate, and the higher pooled result is primarily driven by outlier data from pharmacovigilance reporting.

Evidence recommended that genetics can modulate the risk of QTc during MMT, but only a few variants show reproducible signals. The strongest finding to date is in KCNE1; a candidate gene study identified rs11911509, which remained significantly associated with QTc after correction for multiple testing and showed a gene × dose interaction. QTc was increased only in AA carriers with higher methadone doses.^[46]^ Recently published data also suggest that KCNH2 (human Ether-à-go-go related gene) variants; in a recent cohort of MMT recipients, a synonymous KCNH2 polymorphism was associated with longer QTc, supporting the model of susceptibility to channelopathy in some populations.^[47]^ Pharmacokinetic genes (CYP2B6, ABCB1) reported to influence methadone plasma concentrations consistently, which could indirectly influence QT risk, although direct links to QTc/TdP are less consistent in studies.^[48]^ In contrast, strong genetic predictors of TdP specifically in MMT is still scarce because TdP cases are rare and often confounded by nongenetic factors (e.g., electrolytes, concomitant medications), and most genetic insights have been extracted from congenital/drug-induced LQTS literature (e.g., KCNQ1, KCNH2, KCNE1, SCN5A) rather than MMT cohorts.^[49–51]^

## 5. Conclusion

Our study specifically focused on the occurrence of cardiac complications, namely QT prolongation and TdP, in patients undergoing MMT. Based on our results, MMT is associated with a substantial risk of QTc prolongation and a low but clinically relevant risk of TdP. Careful dose adjustment and regular ECG monitoring, particularly at higher doses, are essential to minimize cardiac complications. Although this study provides useful data on the arrhythmic potential of methadone, further studies are needed to assess its overall efficacy as well as other short- and long-term cardiac complications. Such research would help to more uniformly weigh the therapeutic benefits of MMT against its cardiovascular risks.

## Author contributions

**Data curation:** Mohammad Hossein Paknahad, Atefeh Amerizadeh, Raheleh Karimi.

**Formal analysis:** Mohammad Hossein Paknahad, Atefeh Amerizadeh, Raheleh Karimi.

**Investigation:** Mohammad Hossein Paknahad.

**Methodology:** Mohammad Hossein Paknahad, Raheleh Karimi.

**Project administration:** Azam Soleimani.

**Resources:** Fatereh Baharlouei Yancheshmeh.

**Software:** Parisa Adib-Hajbagheri.

**Supervision:** Azam Soleimani.

**Validation:** Azam Soleimani, Atefeh Amerizadeh.

**Writing – original draft:** Fatereh Baharlouei Yancheshmeh, Parisa Adib-Hajbagheri.

**Writing – review & editing:** Parisa Adib-Hajbagheri, Atefeh Amerizadeh.

## Supplementary Material


