# Supplementary material for: QTc prolongation and torsades de pointes (TdP) in individuals undergoing methadone maintenance treatment (MMT): A systematic review and meta-analysis
Source: Medicine (Baltimore). 2025 Oct 24;104(43):e45304. doi: 10.1097/MD.0000000000045304 (PMC12558210; doi:10.1097/MD.0000000000045304)
Supplement: Supplementary file 1 [file medi-104-e45304-s001.docx]

**Appendix 1: detailed search strategy in main databases**

Here’s a detailed search strategy for identifying studies on QTc prolongation and Torsades de Pointes (TdP) in methadone users in major databases like PubMed, Embase, and Web of Science. The strategy should include specific keywords, Medical Subject Headings (MeSH), and filters tailored to each database.

### 1. **PubMed Search Strategy**

#### Search Query:

1. **Methadone-related keywords:**

- "Methadone"[MeSH] OR methadone OR "methadone maintenance" OR "methadone treatment"

2. **QTc prolongation-related keywords:**

- "QTc Interval"[MeSH] OR "QTc prolongation" OR "QT interval" OR "prolonged QT" OR "QT prolongation"

3. **Torsades de Pointes-related keywords:**

- "Torsades de Pointes"[MeSH] OR "torsade de pointes" OR TdP

4. **Combine with AND:**

- (Methadone) AND (QTc prolongation OR QT interval) AND (TdP OR Torsades de Pointes)

5. **Filters:**

- Articles published up to September 2023

- Language: English

- Study Types: Observational studies, cohort studies, case-control studies, cross-sectional studies

#### Full Example:

`("Methadone"[MeSH] OR methadone OR "methadone maintenance" OR "methadone treatment") AND ("QTc Interval"[MeSH] OR "QTc prolongation" OR "QT interval" OR "prolonged QT" OR "QT prolongation") AND ("Torsades de Pointes"[MeSH] OR "torsade de pointes" OR TdP)`

### 2. **Embase Search Strategy**

#### Search Query:

1. **Methadone-related keywords:**

- ‘Methadone’/exp OR methadone OR "methadone maintenance" OR "methadone therapy"

2. **QTc prolongation-related keywords:**

- 'QTc Interval'/exp OR "QT interval" OR "QT prolongation" OR "prolonged QT"

3. **Torsades de Pointes-related keywords:**

- ‘Torsades de Pointes’/exp OR "torsade de pointes" OR TdP

4. **Combine with AND:**

- (Methadone) AND (QTc prolongation OR QT interval) AND (TdP OR Torsades de Pointes)

5. **Filters:**

- Articles published up to September 2023

- Study Types: Observational studies, case reports, cohort studies

#### Full Example:

`('methadone'/exp OR methadone OR 'methadone maintenance' OR 'methadone therapy') AND ('QTc Interval'/exp OR 'QT interval' OR 'QT prolongation' OR 'prolonged QT') AND ('Torsades de Pointes'/exp OR 'torsade de pointes' OR TdP)`

### 3. **Web of Science Search Strategy**

#### Search Query:

1. **Methadone-related keywords:**

- TS = (methadone OR "methadone maintenance" OR "methadone treatment")

2. **QTc prolongation-related keywords:**

- TS = ("QTc prolongation" OR "QT interval" OR "prolonged QT" OR "QTc interval")

3. **Torsades de Pointes-related keywords:**

- TS = ("Torsades de Pointes" OR "torsade de pointes" OR TdP)

4. **Combine with AND:**

- (TS = (methadone)) AND (TS = ("QTc prolongation" OR "QT interval")) AND (TS = ("Torsades de Pointes" OR TdP))

5. **Filters:**

- Publication date: Up to September 2023

- Document Type: Article, observational study, case-control study, cohort study

#### Full Example:

`TS = (methadone OR "methadone maintenance" OR "methadone treatment") AND TS = ("QTc prolongation" OR "QT interval" OR "prolonged QT") AND TS = ("Torsades de Pointes" OR "torsade de pointes" OR TdP)`

### Key Notes:

- **Adjust keywords for specificity: ** Depending on your research question, you can add additional synonyms or narrow down specific drug formulations.

- **Study Types: ** Make sure to use filters for observational studies, as they are the most relevant for your meta-analysis.

- **Language and Date Filters: ** Apply filters to retrieve studies only in English and published up to September 2023.

This strategy ensures comprehensive coverage across the three databases for identifying studies on QTc prolongation and TdP among methadone users.
