# Supplementary material for: QTc prolongation and torsades de pointes (TdP) in individuals undergoing methadone maintenance treatment (MMT): A systematic review and meta-analysis
Source: Medicine (Baltimore). 2025 Oct 24;104(43):e45304. doi: 10.1097/MD.0000000000045304 (PMC12558210; doi:10.1097/MD.0000000000045304)
Supplement: Supplementary file 2 [file medi-104-e45304-s002.docx]

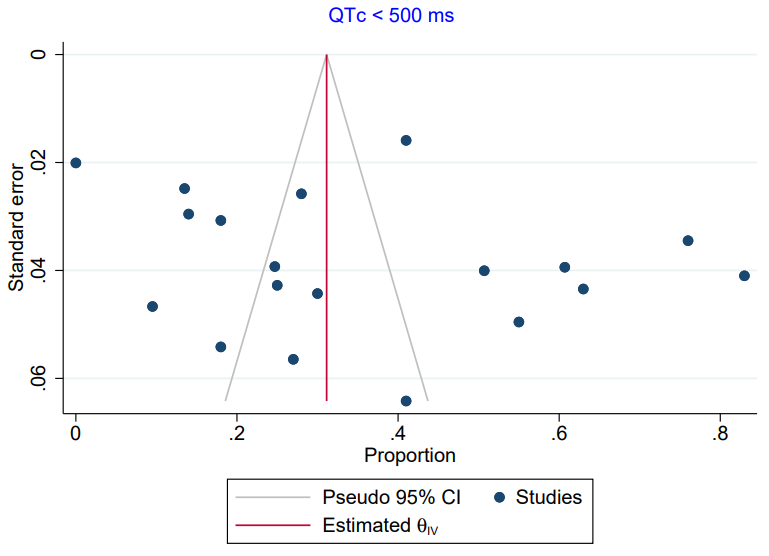


p-value (Egger test)= 0.405

# [Funnel plot for the prevalence of QTc prolongation > 450 ms and <500 ms after MMT](https://www.researchgate.net/figure/Funnel-plot-for-the-association-between-garlic-consumption-and-colorectal-cancer_fig1_276071728)


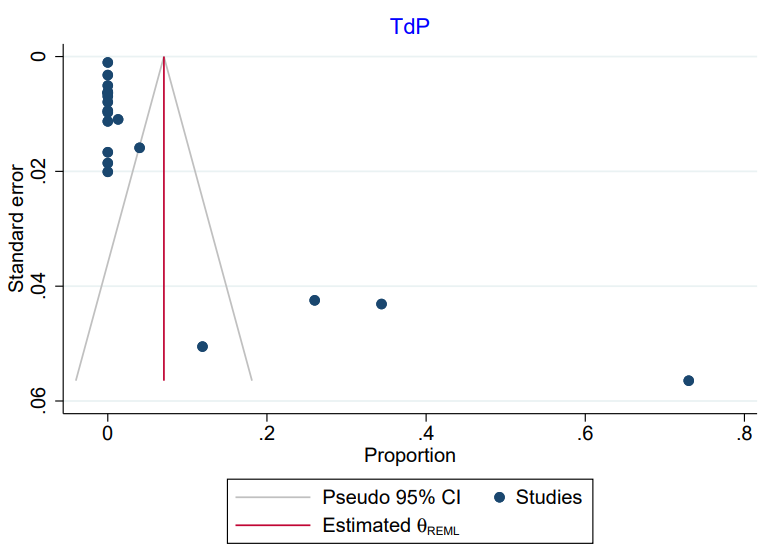


p-value (Egger test) < 0.001


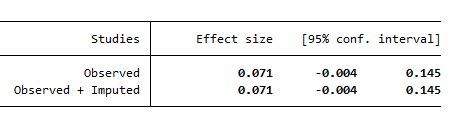


# Funnel plot for the prevalence of TdP after MMT


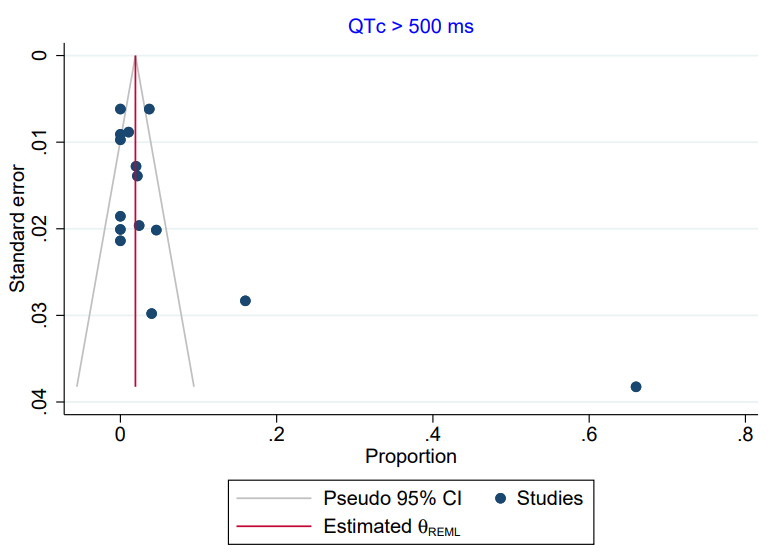


p-value (Egger test) < 0.001


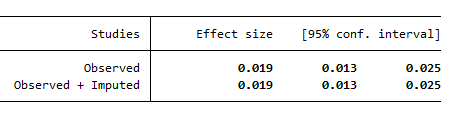


# Funnel plot for the prevalence of QTc prolongation > 500 ms after MMT


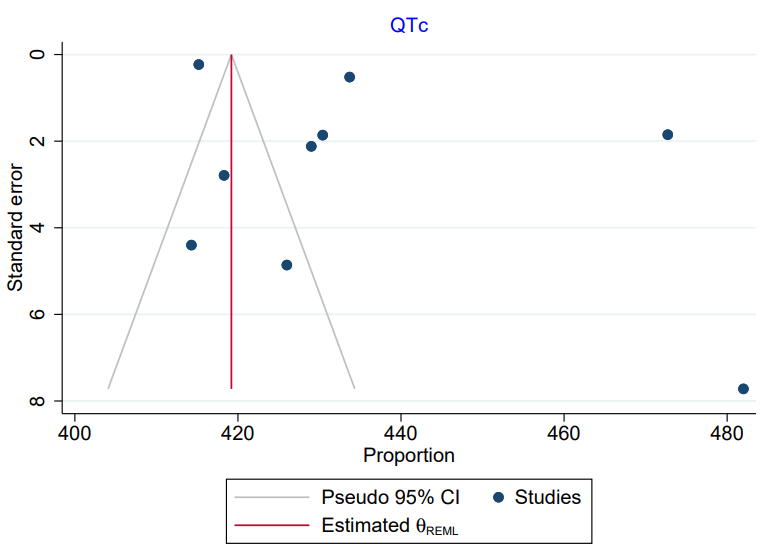


p-value (Egger test) = 0.220

# Funnel plot for the mean of QTc after MMT
